# ‘*We Call it Jail Craft*’: The Erosion of the Protective Discourses Drawn on by Prison Officers Dealing with Ageing and Dying Prisoners in the Neoliberal, Carceral System

**DOI:** 10.1177/0038038517695060

**Published:** 2017-04-07

**Authors:** Marian Peacock, Mary Turner, Sandra Varey

**Affiliations:** Manchester Metropolitan University, UK; Lancaster University, UK; Lancaster University, UK

**Keywords:** end of life, inequality, neoliberalism, nostalgia, prison officers, prisons

## Abstract

The UK prison population has doubled in the last decade, with the greatest increases among prisoners over the age of 60 years, many of whom are sex offenders imprisoned late in life for ‘historical’ offences. Occurring in a context of ‘austerity’ and the wider neoliberal project, an under-researched consequence of this increase has been the rising numbers of ‘anticipated’ prison deaths; that is, deaths that are foreseeable and that require end of life care. We focus here on ‘jail craft’; a nostalgic, multi-layered, narrative or discourse, and set of tacit practices which are drawn on by officers to manage the affective and practical challenges of working with the demands of this changed prison environment. Utilising findings from an empirical study of end of life care in prisons, we propose that the erosion of jail craft depletes protective resources and sharpens the practical consequences of neoliberal penal policies.

## Introduction

Neoliberalism has been shown to have negative health and social consequences across a wide variety of domains (De Vogli, 2011; Navarro, 2007). However, the mechanisms via which neoliberal discourses and practices cause damage or erode protections need to be both more precisely specified and better understood in order to tease apart the well-established consequences of life in unequal societies from those we are proposing connect specifically with neoliberalism (Wilkinson and Pickett, 2009). Which neoliberal practices and discourses might sharpen the damages of inequality and how these might be manifest, require exploration in order to avoid what Bell and Green (2016: 239) have described as ‘the perils of invoking neoliberalism in public health critique’; that is, using neoliberalism as a catch-all term that sheds no further light on underpinning processes. In order to do this, we look here at a set of discursive and affective aspects of contemporary neoliberalism encountered in the prison as a workplace. We focus on the consequences of recent changes within the prison system and the expansion of neoliberal governmentality, and consider what this means for the protective mechanisms and resources that can be drawn upon in the context of work. To illustrate these processes we examine a key theme in the findings from an empirical study of end of life care in prisons.

How the practical and policy changes associated with neoliberalism might engender health and social problems may be considerably easier to understand than the discursive or affective aspects. These practical changes include the transfer of public goods to the private sector – what Esping-Andersen (1990) calls ‘recommodification’, a widespread reduction in social provisions such as welfare benefits and social housing, and a widening income gap across societies. The ensuing increased poverty and inequality has been shown to have a wide range of health consequences (Navarro, 2007). These changes also have consequences that go beyond the material, with the transfer of risk onto individuals and the rise of precarity and uncertainty, and have been shown to have differential effects across the social gradient, with the poorest faring worst (Standing, 2011). But discourses, cultural practices and what Williams (1977) has called ‘structures of feeling’ are also shaped by neoliberalism and in turn serve to shape a neoliberal subjectivity and identity in ways that can have additional damaging consequences for health and well-being (Rose, 2001).

As Wacquant (2010) and others have shown (Esping-Andersen, 1990; Wilkinson and Pickett, 2009), the extent to which countries have embraced neoliberal/market perspectives predicts negative health and social consequences and correlates with increasing prison populations. What Wacquant has termed the ‘neoliberal penal surge’ and the consequent growth in the ‘carceral state’ has resulted in burgeoning prison populations, notably in the US, but with the UK being the European country most closely following the US penal model. An unintended but striking feature of this increase in incarceration is the growing number of older inmates and the associated increase in anticipated deaths as people age and die in prison, are sentenced when already old and, for both older and younger terminally ill prisoners, are refused compassionate release or release on licence.

In the UK, palliative and social care beds in prisons are few in number and provision across the prisons estate is patchy. In some locations, high quality palliative care is available, but whether or not there is specialist provision, staff are increasingly likely to find themselves working with prisoners in the last months or year of their life. While the brunt of these changes has been borne by prisoners, there are also significant and under-researched consequences for staff. As Crawley has argued:

While the sociology of the prison has acknowledged the impact of prison on the emotional lives of prisoners … there has been much less academic interest in the emotional impact of the prison on its uniformed staff. (Crawley, 2004: 412)

The study that we are drawing upon to develop our argument is ‘*Both sides of the fence: using action research to improve end of life care for prisoners*’ (Turner and Peacock, 2017). This article focuses on one aspect of the wider study findings for prison discipline staff (prison officers), looking at the impacts that neoliberal, carceral policies have on their capacities to manage this new and challenging area of work. We examine the resources available for officers to draw upon to protect themselves, how these impact on practices, how the loss of protection serves to increase insecurity both practically and as embodied and felt, and what these changes mean for the wider prisons system.

To do this we are focusing on what officers term ‘jail craft’, a discourse which emerged in interviews and focus groups as officers struggled with the changing nature of the work that they were expected to undertake. Jail craft is a multi-layered narrative or discourse, and a set of tacit practices which allow officers to maintain order and have functioning working relationships with prisoners, where authority and respect are maintained and where the self is protected, in part, from the vagaries of prison life. It is not just the individual self: jail craft can only be understood when considered as a collective enterprise, rooted in history and enacted in the present. Jail craft resonates with a broader literature on work and work identities (Sennett, 1998; Strangleman, 2006), connecting with skill, pride and notions of solidarity. We propose that this is in part a nostalgic discourse, but that this nostalgia can help to better understand the present, and that collective nostalgia is part of a wider range of resources used to protect the self. While the focus of this article is prison officers, some prisoners also use versions of this discourse to attempt to recapture a world of clear identities and of mutually maintained boundaries.

History and point in time are also important here; the study was conducted while the ‘benchmarking’ process in the UK prison service, a neoliberal austerity measure, was being rolled out. Benchmarking led to the greatest reduction in prison staff numbers (30%) in the history of the prison service in England and Wales (Parliamentary Justice Committee, 2016). This loss of experienced officers and subsequent press coverage of increased violence (including murder) and a range of discipline and safety problems then led to 2000 officers who had retired or been made redundant being offered fixed term contracts (across England and Wales) to ensure safe staffing levels (Prison Reform Trust, 2015). Unsurprisingly, the practical strains on staff have increased and, we propose, there has been an erosion of the collective and pride-driven discourse of jail craft as a source of protection for officers from the challenges of an ageing and ill prison population.

To better understand these changes we now examine the changed prison population in more detail.

## The Neoliberal Penal Surge and the Changing Prison Population

In England and Wales (Scotland has a separate system) the prison population has doubled in the last decade to 85,106, with the numbers of prisoners over 60 having tripled (Ministry of Justice (MOJ), 2015); the numbers of the ‘oldest old’ (over 85 years of age (Turner and Peacock, 2016)) increasing most dramatically. By March 2014 there were more than 100 people in prison over the age of 80, with five being 90 or older (Prison Reform Trust, 2015). At the time of writing, a 101-year-old man has been sentenced to 13 years for historical sexual offences. It is important to note that this expansion of prisoner numbers has occurred over a time period when crime rates have been either flat or falling and is a result of longer sentences, a greater willingness to imprison older people and more restrictive licensing conditions resulting in greater numbers of recalls to prison. As well as these legislative changes, there has been a shift in attitudes to certain kinds of crime; in the UK this is primarily what has come to be known as ‘historical sexual abuse’, with courts showing a greater willingness to believe victims, more victims coming forward (Gray and Watt, 2013) and sentence duration increasing. The resultant older prisoner population means more and increasing deaths due to natural causes. The overwhelming majority of these prisoners are male and over 40 per cent are sex offenders.

In 2014, 107 prisoners over the age of 50 died of natural causes (MOJ, 2015) and, although some deaths may have been sudden and not possible to anticipate, the majority of deaths followed long periods of ill-health entailing a need for palliative or end of life care. Contrary to what many believe, compassionate release in the event of a terminal diagnosis is rare, with only 45 people being released between 2009 and 2013 – less than a tenth of the numbers of natural deaths in prison over this time (Hansard, 2014). Prison staff, and in particular officers who work on the residential wings (those who have the most consistent daily contact with prisoners), are now working in conditions and with populations that are very different from those of the past and which make new and difficult demands upon them; it is to this that we now turn.

## The Prison as a Workplace

Changes in the nature of work and workplace relationships have characterised the advance of the neoliberal project and it is in the workplace that new neoliberal identities can be forged. But the workplace is also the location of narratives and practices which can protect against the vagaries of neoliberalism and be sources of social resilience (Hall and Lamont, 2013). As Gammon argues: ‘to understand neoliberalism we need to grasp the way it is grounded in concrete social relations and lived practices’ (2012: 519). In the case of the prison service, it is well known that prison officers experience high levels of stress (Johnson et al., 2005) and rising rates of sickness – more than double the population average (Prison Reform Trust, 2015). Prisons are frequently at the centre of political debates and what happens, or is believed to happen, there can be deployed as a rhetorical tool to legitimise a range of political decisions and policies. It is not, therefore, surprising that prison and prison discourses are connected to the wider neoliberal project.

Prison officers have to find ways of conducting themselves that manage the well-known tensions between care and custody (Turner et al., 2011), tensions that are exacerbated by the altered prison population of the old and the frail. Aspects of this work can be described as ‘emotional labour’ (Hochschild, 1990: 118), where the individual is required to try ‘to feel the right feeling for the job’. Such emotion work, Hochschild has proposed, may be ‘toe’ or ‘heel’, with ‘toe’ work demanding the enactment of emotions that are more benign than might be reasonably expected and where the goal is to make the recipient feel better than they otherwise might (airline stewards, for example). ‘Heel’ work, by contrast, requires ‘nastier than natural’ ways of being (Hochschild uses the example of debt collectors). Prison officers in the contemporary prison system are in the invidious position of both types of emotional labour being demanded, particularly in the domain of work with dying offenders.

This new world of work is not simply passively accepted but is contested and resisted, both practically and discursively, with protests and strikes about prison conditions and via the ‘struggle for memory’ described by Strangleman (2007: 96). Drawing on Linkon and Russo’s (2002) work around the mass steel closures in the US, Strangleman shows the importance of collective memories of resistance and pride in the past for resisting distortion and denial of agency and collectivity in the present, and how these can form a resistant resource. It is these sorts of resources that are being depleted and compromised by neoliberal ‘benchmarking’.

The ‘the nature of power’ (Crewe, 2011: 456) within prisons has changed enormously, with policies now being ‘predominantly neo-liberal in their character’. By this he means a form of ‘power at a distance’ which operates ‘via self-interest and self-regulation … less directly coercive or authoritarian than in the past … however it grips tightly, constrains effectively and is highly intrusive’. This ‘soft power’ is enacted in the new staff–prisoner relationships, which are a key aspect of the ‘dynamic security’ central to the contemporary prison service. Coercion or ‘hard power’ are seldom necessary, but are there to be used when necessary, with prison officers and prisoners encouraged to relate in ways very different to the screw/con divide of the past. As Crewe puts it:

These policies encourage prisoners to regulate their own behaviour, putting the onus on them to govern their conduct, address their offending behaviour, engage positively with the regime and accept responsibility for any failings to do so. They are predominantly neo-liberal in their character; aspects of what Garland (1997) and others refer to as ‘governmentality’. (Crewe, 2011: 456)

‘Dynamic security’ is contingent on the nature of relationships between officers and prisoners as well as on adequate staff, and for officers who are now working with old, disabled, frail and dying prisoners, this aspect of neoliberal governmentality has troubling consequences. Firstly, it places officers in close proximity to suffering and dying, often with prisoners who are atypical, primarily due to the imprisonment of men who are already old for the first time for ‘historical’ offences. These prisoners are resident on wings for ‘vulnerable prisoners’ (VPs) and do not present the sorts of challenges to order and discipline found in the ‘mains’ (non-VP) prison population.

Secondly, this is happening in prisons that have experienced major cuts in resources and a reduction in the numbers of senior and experienced officers, against the backdrop of a growth in private prisons, which many officers in public sector prisons see as threats to their professional status and income. Heslop (2011) has described the ‘McDonaldization’ of the British Police Service and there are concerns among prison officers of a similar process of the stripping away of autonomy and increases in fragmentation and external control.

## The Study

The aim of the study was to improve palliative and end of life care in prisons. The World Health Organization defines palliative care as ‘an approach that improves the quality of life of patients and their families facing the problem associated with life-threatening illness, through the prevention and relief of suffering’ (Hall et al., 2011: 6), with end of life care usually being understood to apply to the latter stages of this process. Ethical and governance approvals were obtained from Lancaster University, the NHS and the National Offender Management Service (NOMS) and a single prison with a high proportion of older, male, chronically ill and disabled prisoners was selected as the study site. In the first phase of the study, we ran focus groups and conducted individual interviews (*n* = 62) both inside and outside the prison with prison staff, prisoners, palliative care specialists and others. All the focus groups were private with no management present in the officers’ groups and no officers in the case of the prisoner groups. In addition, we undertook a range of informal conversations and observations across the prison, including attending meetings and shadowing key staff on both day and night shifts. All the interviews and focus groups were transcribed in full, reflective field notes were made and a range of documents relating to end of life care collected. All of these data were analysed thematically within NVivo 10 Software, using the conceptual and empirical framework of ‘thematic networks’ (Attride-Sterling, 2001).

## Findings

This first phase of the study scoped and explored current practice around end of life issues in the study site, including the thoughts and feelings of staff and prisoners about proposed changes in relation to end of life care provision within the prison itself. At this point the prison was planning to convert a cell into a specialist palliative care facility, similar to those found in a minority of UK prisons. A number of anxieties and frustrations from prison officers emerged about work they felt was being thrust upon them in relation to older and dying prisoners. There was a high level of concern about what a dedicated palliative care facility and an increase in frail, ill and disabled prisoners would mean for them in the future. It was in the course of these discussions that the discourse of jail craft emerged.

The quotes below are from prison discipline staff. These include officers (main grade staff), custodial managers (CMs, who are more senior and experienced) and governors (non-uniform, managerial grades). Participants are numbered and grade of post indicated to allow the reader to understand who is speaking and the range of voices.

### Jail Craft – An Introduction

As prison officers struggled with how to manage the new practical constraints and their own feelings concerning these changes, they drew upon a variety of discourses that served as partial protections from the new stressors. These discourses contain traditions, some of which may be double-edged, as studies of prison officers tend to show discourses of pride, defensiveness and sometimes a siege-like mentality (Liebling, 2011), which are reflected in the discourse of ‘jail craft’, the focus of this article:

We call it jail craft. And you’ve got to learn that as jail craft … there’s a balance in it, you know. You’ll always find anyway that the roughy-toughies, let’s say, eventually the penny drops because prisoners will not react to them … and eventually they think ‘Oh well, hang on a minute, I need to change tactics’. (Governor 02)

Another governor contrasted those with good jail craft and those without:

They’re the ones with the big muscles … they walk round and that’s the way they live their life. You get the other prison officers that are just the opposite; never do any of that … but if it came down to ‘hey lads hey’, they can still handle their selves and … a lot of them will have the better jail craft. Now I’m not saying all the big lads don’t, but [the ones with jail craft] have the compassion and they’ll have the decency to treat the prisoners properly. (Governor 03)

Good jail craft can facilitate work with a changing prison population and protect the officer’s professional identity in that process. However, at the point in time when our study was conducted, aspects of jail craft were experienced as being under attack and there was an impending loss of a significant cadre of officers who embodied such practices. This had the effect of bringing the practices and discourses around jail craft into sharper focus.

### Costs of Benchmarking: Resources and Frustrations

Over the years we’ve watched the resources dwindle but the expectations become a lot higher … the budget will always dictate the level of care: whether it’s end of life care or any sort of care, the budget will dictate what sort of level of care these prisoners receive. It’s the bottom line really – end of political broadcast. (PO 02, focus group)

The concerns and frustrations that emerged in discussions ostensibly about end of life care arose in the context of the recent changes in the prison service outlined above. Thus, both the timing of the study and contemporary wider political and social change are central to understanding what colours the officers’ speech. Overall there was a sense that these changes had taken a heavy toll on both prison staff and on prisoners:

Yeah, it seems to be expectations: you’ve got more jobs to do and with less … less resources, and there’s going to be more cuts as well, so it’s not going to get any better. (PO 03, focus group)

There was also a sense that the prison service just expected people to get on with it, and that burdens would be further exacerbated by dedicated end of life/palliative facilities:

The Prison Service are renowned for the willing horse syndrome; put it on a person … If it’s a physical discipline thing, ‘Well, such and such’s going to do that because they always do it’. If it’s a touchy-feely thing, ‘Well, such a body’ll do that …’. And you get that person labelled and I worry that if the [palliative care suite] has been set up … that’s what’ll happen. (PO 04, focus group)

These changes also had powerful affective dimensions and generated a turn to nostalgic narratives (which, interestingly, were also drawn upon by an ‘experienced’ prisoner who we interviewed, mirroring the content of the officers’ discourses).

### Prison Officers and End of Life Care

Perhaps surprisingly, given the demands on officers, there was a striking emphasis on a common humanity when it came to end of life care:

I think everybody, no matter what their background is, deserves a level of care, a level of dignity, and their families – they also should be receiving that support. If they were in the community they would be getting it, so what is different? Just because they’re a prisoner, just because they’ve done wrong in life – haven’t we all? (CM 02)

While there were certainly officers who did not hold these views, many did, even if sometimes grudgingly. But there were concerns around such work:

I don’t think we’re trained to deal with that. I mean, the nine weeks I spent [on basic training] I certainly don’t remember anybody talking anything about that. I think we probably had a chat off the chaplaincy for half an hour or so, but I certainly don’t remember anybody touching on sort of end of life stuff. (CM 03)

It was the all-present and all-consuming nature of such contact that was seen as hard; officers are used to ‘bed watching’ (where officers escort and guard an ill or dying prisoner in an external healthcare setting such as a hospital), but the new circumstances were very different and were becoming more so:

I think you’re putting a lot of pressure on the staff who’ll … deal with that. It’s fine for me to go to a bed watch, do a 6–2, empathise with the family, empathise with the guy, come away and I’ve forgot about it … If I’m coming in on a shift and I know I’ve got two lads who are terminally ill and I’m on there for the day, for the week, because you can do a ten, well eight-day stretch that can be quite … quite oppressive, especially if you’ve got your own personal circumstances as well. (PO 05, focus group)

There were also fears about how dying in the prison connected with personal life:

It’s a very sort of, I think, difficult and deep subject to speak to people about, and especially people who’ve sort of already experienced it in their private lives outside may find it difficult. Some people might go, ‘Well, why should we care?’, you know, but it’s part of your job, you know, you’ve got to care whether you want to or not. (PO 06, focus group)

Officers described how they managed these changes and the toll that this takes:

Well, you manage it. I don’t think you block it out, you just manage it, because you can’t block it out, you know, you wouldn’t be human, would you? You’d have no emotions or feelings. (PO 07, VP wing focus group)

Officers had not anticipated this sort of work when they came into the prison service:

[Prisoners now are in] their late 60s, 70s – even now into the 80s … Their needs are different … it’s more around medical, health issues; not really any control problems as you get in the younger population … a lot of family problems because of the offence, if it was committed in the family … How you deal with people as well. I think some of the staff probably find it difficult – because with the younger population it’s more you front it out and shouting, and the older guys you don’t … they don’t need that. (Governor 04, VP wing)

There were some officers who were very experienced in work with this older population who described quite radical and caring ways of being:

Well, … we tend to use first-name terms on here to prisoners and at night-time it’s ‘Good night, God bless’, or ‘Good night, see you tomorrow’. Or it’s … you know, you can laugh … it’s true [this directed towards other officers in the group]. No, but they do like … when you’re closing their door at night-time, because they’re going to be locked in for a lot of hours … and you say, ‘Good night’, nine times out of ten you get, ‘Thanks, Miss, good night’. (PO 08, VP wing focus group)

There was recognition from some prisoners of the challenges that officers faced, as this extract from a mains prisoner shows:

Because, you know, the staff use their shields the same way that everybody else does. They don’t want to think about dying. (Mains prisoner 01)

### Protective Narratives, Jail Craft and Nostalgia

As officers struggled with what end of life issues meant for them, they described the sorts of strategies and narratives that they drew on to manage their circumstances and anticipate the difficulties that the future might bring (we also saw these enacted and embodied in our observational work). Many of those experienced officers who prided themselves on their jail craft were about to leave, with fears of the permanent loss of certain kinds of resources embodied in the exiting, experienced staff:

I mean, it’s across the Service … even myself, I’ve decided to … leave because I’ve just had enough. It’s … I love the people I work with, but I hate the politics of the job, I really do … and I think it’s going to get worse. I couldn’t do this job for another five years … I mean I already take tablets now for my cardiac … and that is down to this job. (CM 02)

Officers turned to memories of the past and how the Service used to be in stark contrast to today:

Prisoners then didn’t tend to sort of argue back with staff. It was, ‘Yes, boss … no, boss, three bags full, boss’ and they just got on with it. They were sort of the old lags, if you want to call them that … they were in jail, they knew what the score was, they got on with it, they did the jail and they went out back on the street. … I mean, these days, the discipline’s gone out of the job. (PO 09, focus group)

There is, arguably, in these accounts, a somewhat implausible feeling akin to that of memories of childhoods with endless summers, but like childhood memories, the stories resonate with contemporary experience and there is value in being able to access them:

There’s no discipline in the Service now. I mean, even down to the staff … I know we’re in sort of this ever-changing world and it’s equal opportunities regardless of your gender, your nature or whatever, but I mean staff walking round with earrings in and stuff … long hair and studs in their eyebrows – I mean back then it was … very much a disciplined service … the POs and the chief officers, they were like gods … they would make sure the job got done, they made sure the staff were looked after … just as I started we were still wearing the old blue uniforms … you still had to wear your cap and stuff like that. (CM 04)

This was mirrored by an experienced prisoner, whose comments also illustrate the sorts of soft power described by Crewe (2011) above:

Years ago it was ‘them and us’ and that was good … because it gave you a place … You could go and talk to the staff … so there was basically the brick wall between us. But all of that’s been taken away now because they rely so much on information received. They want people to be able to let things slip conversationally … You know, there is no boundaries as such … I hear a lot of these young‘uns calling the staff ‘mate’ … wouldn’t have happened years ago. It was ‘Gov’ or ‘Boss’. That’s gone by-the-by now … I call them ‘Mr H’, ‘Mr B’, whatever. That suits me because I’m old-fashioned in that respect … Show you the respect for the job that you’re doing, but you’re a screw, I’m a con. It’s a strange animal at the moment, prison life … it used to be organised, it used to be regimented … but all that’s just slid. There’s not enough staff to govern … anymore. (Mains prisoner 01)

What is happening here, we propose, are forms of moral work or positioning as a part of the sorts of emotional labour described above. This work facilitates ways of being and forms of legitimacy which protect the self. Jail craft and what this represents as memory, tradition and aspiration both structures the permissible forms that this work can take and, with its emphasis on solidarity and what can be expected of others, provides protection. While it is the individual that is undertaking this work, these tasks can only be accomplished in the collective setting and by drawing on shared or collective narratives formed in and around the workplace.

## Discussion

Key aspects of the officers’ discursive and affective world connect with solidarity and collectivity, a picture found in other studies (Crawley, 2012), and it is in these domains in particular that neoliberal discourses are becoming hegemonic. Neoliberalism is the antithesis of collectivity, with neoliberal governmentality actively undermining solidarity, increasing precarity and a growth in individualism (Strangleman, 2012). The occupational culture of the prison service shapes and is shaped by forms of solidarity that can be both a resource and a protection, as well as reactionary or defensive. Studies in the sociology of work have shown that certain occupational groups develop strong occupational cultures, with mining and steelwork being prime examples. A part of these cultures are the collective resources and collective imaginaries which can be drawn upon to protect; even when industries have all but disappeared, these resources remain available at least in part. There tend to be common characteristics of these sorts of work, with MacKenzie et al. (2006: 836) commenting that: ‘Particularly where the work is demanding, dangerous or highly skilled, individuals can develop a strong emotional attachment to their work’; criteria that very much apply to prison officers.

A closer comparator with the prison service is arguably the police, and there are similarities between the two in terms of occupational cultures, particularly around attempts to change reactionary or discriminatory practices; there are also some marked differences. Loftus has described how the dominant culture within the police force resisted attempts to ‘diversify’: to include more women and minority ethnic groups. While the culture was ‘disrupted’ by these new recruits, there remained ‘a resilient residue of dispositions that undermine the requirements of the new terrain’ (2008: 774) and which drew on nostalgic discourses to defend and rationalise this resistance. We would argue that the response of prison officers to the changes detailed above is different to this. In part it may be that current prison changes represent less of a threat to identity and power than diversity is understood to do in the police, but in addition the discourse of jail craft is more akin to a craft narrative like those described by Sennett (1998) and Strangleman (2006), placing centre stage the skills to achieve order without excessive force. The domestic nature of the prison residential wings and the simple numerical calculation of the ratio of officers to prisoners in achieving consent will also play a part. In this context, jail craft and the tacit practices it embodies is understood by officers as an art and craft, learned from those who are more experienced, and is used as a resource or tool. Thus, a nostalgic prison culture of the past is evoked to defend against challenges of the present.

Nostalgia is often assumed to be a conservative or even reactionary emotion with an edited or sentimentalised past being evoked, minimising what was problematic then and damning what is happening in the present. This form of nostalgia is evident in the work of Loftus (2008). However, as Strangleman, in his studies of the rail industry shows, drawing on the work of Fred Davis (1979), rather than being passive or conservative, nostalgia ‘tells us more about the experience of contemporary life than it does about the past’ (Strangleman, 2012: 414). As well as ‘simple nostalgia’, Davis (1979) proposes a reflexive nostalgia; a ‘more complex human activity that can better comprehend our selves and our pasts’. This form of nostalgia goes beyond the sentimentalisation of the past and its corresponding counterpoint of a more negative present. In reflexive nostalgia the past is evoked with the same warmth and yearning as in simple nostalgia, but there is an active questioning and scrutiny of the memories and claims. Within reflexive nostalgia there is another voice or ‘Greek Chorus’, comparable, argues Davis, to the reality testing function of the ego in Freudian theorising. Third order or interpreted nostalgia moves beyond the first two to ‘render problematic the very reaction itself’ (1979: 24) with an analytic stance towards the feeling, questioning why this is being felt now and asking what this might mean for understandings of the past.

Nostalgia is evoked and drawn upon as a resource in the presence of contemporary anxieties, fears or discontents that ‘pose the threat of identity discontinuity’ (Davis, 1979: 34). Modernity and the fast pace of change present constant challenges to identity and require an evaluative stance towards former selves and, as Davis emphasises, a need to think ‘well’ of the self. But, he argues, there are also circumstances that facilitate ‘collective nostalgia’ where the individual experiences of many become forged into a whole; change is experienced collectively rather than in the individual life, creating a collective memory which may protect and spur action in the present. Collective nostalgia, Davis proposes, is evoked by major social change: ‘wars, depressions, massive natural disasters, death and assassinations’ (1979: 102). In this article we are arguing that in the contemporary case of the prison service, there is a more localised form of collective nostalgia, shaped by a shared workplace history and traditions, and evoked in the present by the severe threats to identity and to the nature of work that benchmarking and the growth in anticipated deaths in prison represent.

Strangleman has argued (1999) that nostalgia and a contestation of the past can also be consciously used by management to ensure ‘ontological insecurity’ in a workforce with the aim of being better able to push through or maintain undesired change. An alternative past is created, one ‘not experienced by the workforce themselves’ (1999: 742), and it is this past that shapes a vision of the future. In recent times, UK prisons and the governance of prisons has had a higher and considerably more negative profile than the current government would probably have wished. Sections of the prison service have been privatised with the rationale for privatisation, and for the benchmarking process described above, resting on a narrative of public sector inefficiency and trades unions as an outdated obstacle to change; a feature of much of the expansion of the neoliberal project. This use of a nostalgic counter-narrative is contested in part by prison officer representatives explicitly linking the riots, increasing drug use and deaths to the loss of experienced officers and, by implication, the loss of the narrative of jail craft.

Thus, the content of the narratives and the extent to which they are invested in can tell us not just about the past but about what is happening in the present that may be shaping these narratives. As Strangleman (2012: 423) has argued: ‘The task for sociology is to understand what lies behind these narratives of decline and not to dismiss them as either nostalgic false consciousness or as private troubles which statistics do not recognize as public issues.’ Raymond Williams’ (1977) ‘structures of feeling’ can also help here, a key feature of which is that it is only as they are disappearing that we can see and understand them and their relevance. These ‘residual’ structures of feeling, ‘ways of seeing and being in the world that is gradually being eclipsed, but nonetheless still provides an interpretive framework for those who still value it’ (Strangleman, 2012: 415), can help to make visible what it is that is about to be lost.

How these narratives may operate protects against the invidious consequences of neoliberalism, and specifically against some of the well-known negative consequences for health described by Hall and Lamont:

Scripts about collective identity are some of the main repertoires or toolkits on which individuals draw on to gain recognition and respond to the challenges they face … resilience is maintained not only by inner moral strength and resourcefulness or by social support … but also through the repertoires that sustain recognition … or positive conceptions of collective selves. (Hall and Lamont, 2013: 135)

In earlier work, Lamont (2000) described how the better health of working class French men compared to their US counterparts was in part due to more collectivist orientations among the French, which minimised damage, unlike the individualist narratives of those in the US. Similarly, Bouchard (2009) found that the health of Canadian First Nation people with strong adherence to their cultural narratives was better than those with weaker or absent narratives. Where narratives and traditions have been destroyed, the opposite picture prevails, as Collins and McCartney (2011: 501) show in the case of the ‘Scottish effect’; the excess of mortality and morbidity in the West of Scotland compared with equally deprived areas elsewhere in the UK due to ‘neoliberal shock treatment’, as a form of ‘political attack’. Political attack is the conscious destruction of workplaces, trades unions and traditions, in part as a ‘side effect’ of the neoliberal project but also, they argue, consciously as a part of advancing this project. The resultant despair, disempowerment and destruction of protective narratives have psychosocial consequences that connect political attack with adverse health consequences.

Neoliberal discourses, while arguably hegemonic, do not just bear down from above; individuals, groups and communities resist, both explicitly and tacitly, and grasping the nature of this resistance and the resources upon which it is contingent is an important task for sociology. In the prisons, ‘jail craft’ forms part of these resistant resources, and the weakening of jail craft as a collective narrative erodes a work identity that allows for a degree of humanity and dignity in prison governance; it serves to protect the health of members of staff and, by proxy, the health of prisoners. It can help to hold back the imposition of difficult working circumstances, neoliberal governance and the enactment of the neoliberal project. When this discourse is eroded, as it is in the present due to the loss of staff who embody and carry forward the discourse and associated traditions, what is left are individuals (officers) faced with other individuals (prisoners) who are a source of conflict and distress because of their offences and their ailing bodies. The dramatic increases in chronic ill-health, frailty and dying that characterise the contemporary prison service are being experienced firstly by prisoners but also by staff who are losing protective resources at the point when the nature and extent of the demands upon them are at their greatest.

## Conclusion

The Prisons and Probation Ombudsman’s recent report ‘*Learning Lessons Bulletin: Fatal Incidents Investigations*’ (February 2016) identified 39 deaths between 2013 and 2015 from the use of ‘legal highs’ in prisons – a marked increase over the preceding period. This finding figured widely in the press at the time of publication. However, over the same period, more than 200 prisoners died from ‘natural causes’, with an unknown proportion of these deaths needing palliative or end of life care; five times the deaths from legal highs. Legal highs present considerable discipline and good order challenges to prisons that elderly, dying prisoners do not. Indeed, the thematic review of older prisoners’ needs undertaken by HM Inspectorate of Prisons in 2004 reflected this in its title ‘*No Problems – Old and Quiet: Older Prisoners in England and Wales*’; the title being a quotation from the notes of a prison officer.

The increase in prison dying is largely an unintended but entirely foreseeable consequence of a range of sentencing changes and shifts in attitudes consistent with a neoliberal prisons policy. In this article we have focused on staff and on the resources that they draw upon to manage these changed and challenging workplace circumstances as it is primarily prison officers who are dealing with the consequences of such decisions. The empirical study we are drawing on was based in a single prison and, while much of what we are detailing is common across many prisons, there are also numerous specifics which need further research. Higher security prisons and prisons for women, for example, are likely to raise additional and particular challenges. But what is shared is the increase in incarceration of older people, the longer sentences, the lack of appropriate services for those who are dying, the sweeping cuts in the prison service, and consequent increase in sickness and strain in officers and prisoners. All are aspects of the neoliberal project, but neoliberalism is ideological as well as practical, and destroying the stories of solidarity are necessary in order to construct the new neoliberal subjectivity resulting in the individualising of pain, suffering and resistance (Rose, 2001; Standing, 2011). Jail craft as a narrative is not just an uncritical romanticising of the past; it reflects the concerns of the present and is a resource to be drawn upon. The destruction of such narratives is not reducible to a side effect of practical change; it is in part a desired outcome of the neoliberal project as it undermines the solidarity that is anathema to neoliberalism. Bourdieu has described this new neoliberal era as the ‘institution of insecurity’ and ‘domination through precariousness’ (Bourdieu, 2003: 29, cited in Strangleman, 2007) and the contemporary prison exemplifies this. The multiple ethical and practical challenges raised by a growing older prisoner population and the associated rise in anticipated deaths are largely a product of neoliberal policies and governance, and can be best understood, critiqued and addressed through this lens.
